# Robust motion correction in the frequency domain of cardiac MR stress perfusion sequences

**DOI:** 10.1186/1532-429X-15-S1-P41

**Published:** 2013-01-30

**Authors:** Vikas Gupta, Martijn Van de Giessen, Hortense Kirisli, Sharon Kirschbaum, Rob J van der Geest, Wiro J Niessen, Boudewijn Lelieveldt

**Affiliations:** 1 Division of Image Processing, Leiden University Medical Center, Leiden, the Netherlands; 2 Department of Intelligent Systems, Delft University of Technology, Delft, the Netherlands; 3 Biomedical Imaging Group Rotterdam, Erasmus MC Rotterdam, Rotterdam, the Netherlands; 4 Department of Radiology and Cardiology, Erasmus MC Rotterdam, Rotterdam, the Netherlands; 5 Quantitative Imaging Group, Delft, Delft, the Netherlands

## Background

To detect perfusion abnormalities at an early stage of CAD, myocardial perfusion is often assessed by analyzing cardiac MR perfusion (CMRP) images. A combination of rest and stress-induced perfusion allows assessing the ability of the heart to adapt to physical exercise, quantified as the myocardial perfusion reserve index (MPRI).

**Figure 1:**
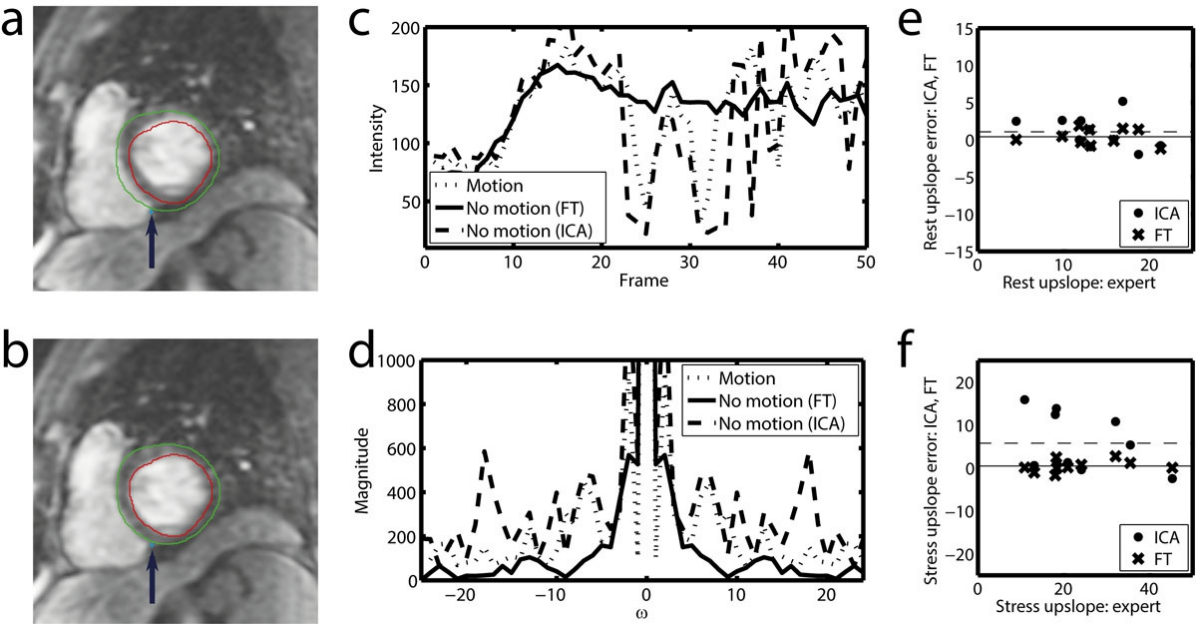
Typical frames of cardiac sequences before a) and after b) motion correction (with FT). The frames are annotated with epi- and endocardial contours. + (near arrows) indicates the annotated displacement evaluation point. (c) Example time-intensity curves in time domain and d) in the frequency domain. The motion artifacts visible in the time domain show up as additional high-frequency content in the frequency domain. e,f) Bland-Altman plots with ICA and FT estimates of relative upslopes in e) rest and f) stress sequences, compared to expert annotations. The means are denoted with a dashed line for ICA and a solid line for FT. For clarity, confidence boundaries have been left out.

However, especially in stress MR acquisitions, the inability of a patient to breath-hold may lead to misalignments between subsequently acquired frames (e.g. Figure 1a) and MPRI, which is based on dynamic contrast uptake (upslope), cannot be measured reliably (e.g., the profile in Figure 1b). Here, we propose a novel motion correction method which is especially aimed at robustness.

## Methods

Motion artifacts manifest themselves as sudden intensity changes over time and show up as high frequency content (Figure 1d). We propose to minimize this high frequency content directly by translating all the frames in the sequence, thereby removing the motion artifacts. The content of the imaged frames themselves does not change. Therefore the original image content is preserved.

A dataset comprising rest and stress images (MRI, 1.5 Tesla) from 10 patients with suspected CAD was used to validate the proposed motion correction method. The registration accuracy of the method was assessed based on annotated myocardium contour locations (Figures 1a and c) and clinically relevant parameters (relative upslope, MPRI). These parameters were based on expert annotations, after motion correction with the proposed method and with an existing method based on independent component analysis (ICA).

## Results

Mean displacements in the non-registered sequences were 2.46 (rest) and 4.85 (stress) pixels (average pixel size: 1.52 mm isotropic). For the proposed method (FT), these decreased to 0.15 and 0.23 pixels, respectively. However, for the ICA based method these were about 1.76 and 5.08 pixels, an motion increase for the stress sequences.

Rest and stress upslope parameters of the proposed method (FT) and the ICA method were compared to expert annotations in Bland-Altman plots (Figure 1e and f) and showed good agreement between FT and expert (not statistically significantly different, level P<0.05), while ICA and experts tended to agree less (P=0.026). ICA mainly failed on stress sequences with large motions. MPRI values showed good agreement between FT and experts (Table 1).

**Table 1:** MPRI values for expert annotation and after FT motion correction

Patient	1	2	3	4	5	6	7	8	9	10
Expert	1.11	4.04	3.84	1.72	1.53	1.52	1.39	2.73	1.24	0.62
FT	1.07	3.65	3.31	1.73	1.58	1.58	1.68	2.55	1.15	0.60

## Conclusions

With minimal user intervention (ROI selection in 1 frame), sequences of 50 frames can now be registered automatically in 20 seconds compared to approximately 1 minute required by ICA and 10 minutes required for manual annotation, while robustly determining upslope and MPRI.

To our knowledge, the minimal user effort, combined with the robustness of the proposed method make it feasible for the first time to process stress sequences in a clinical setting and use parameters such as MPRI in patient care.

## Funding

Agentschap NL, "Heart in 3D" project

